# Effects of early-life exposure to THIP on phenotype development in a mouse model of Rett syndrome

**DOI:** 10.1186/s11689-016-9169-2

**Published:** 2016-10-19

**Authors:** Weiwei Zhong, Christopher Mychal Johnson, Yang Wu, Ningren Cui, Hao Xing, Shuang Zhang, Chun Jiang

**Affiliations:** Department of Biology, Georgia State University, 100 Piedmont Avenue, Atlanta, GA 30302-4010 USA

**Keywords:** *MECP2*, Rett syndrome, THIP, Gaboxadol, Behavior, Locus coeruleus

## Abstract

**Background:**

Rett syndrome (RTT) is a neurodevelopmental disorder caused mostly by disruptions in the *MECP2* gene. *MECP2*-null mice show imbalances in neuronal excitability and synaptic communications. Several previous studies indicate that augmenting synaptic GABA receptors (GABA_A_Rs) can alleviate RTT-like symptoms in mice. In addition to the synaptic GABA_A_Rs, there is a group of GABA_A_Rs found outside the synaptic cleft with the capability to produce sustained inhibition, which may be potential therapeutic targets for the control of neuronal excitability in RTT.

**Methods:**

Wild-type and *MECP2*-null mice were randomly divided into four groups, receiving the extrasynaptic GABA_A_R agonist 4,5,6,7-tetrahydroisoxazolo[5,4-*c*]pyridin-3-ol hydrochloride (THIP) and vehicle control, respectively. Low-dose THIP was administered to neonatal mice through lactation. RTT-like symptoms including lifespan, breathing, motor function, and social behaviors were studied when mice became mature. Changes in neuronal excitability and norepinephrine biosynthesis enzyme expression were studied in electrophysiology and molecular biology.

**Results:**

With no evident sedation and other adverse side effects, early-life exposure to THIP extended the lifespan, alleviated breathing abnormalities, enhanced motor function, and improved social behaviors of *MECP2*-null mice. Such beneficial effects were associated with stabilization of locus coeruleus neuronal excitability and improvement of norepinephrine biosynthesis enzyme expression.

**Conclusions:**

THIP treatment in early lives might be a therapeutic approach to RTT-like symptoms in *MECP2*-null mice and perhaps in people with RTT as well.

## Background

Rett syndrome (RTT) caused mostly by disruptions in the *MECP2* gene is a neurodevelopmental disorder occurring in 1/10,000 live female births [1]. One of the major consequences of the *MECP2* disruption is brainstem dysfunction. [2–5]. In *MECP2*-null mice, several groups of brainstem neurons including those in the locus coeruleus (LC) show increased membrane excitability. As a result of the excessive neuronal excitability, the balance of excitation and inhibition in local neuronal networks is impaired, affecting normal brainstem functions for breathing control, cardiovascular regulation, gastrointestinal activity, arousal, and locomotion, consistent with RTT manifestations in humans [3, 6].

The increased neuronal excitability in the brainstem is attributable to abnormal intrinsic membrane properties and deficiency in GABAergic synaptic inhibitions [7–11]. In *MECP2*-null mice, both GABA_A_ and GABA_B_ synaptic currents are reduced in LC neurons [9]. In contrast, our recent studies indicate that extrasynaptic GABA_A_ currents are well retained in LC neurons of *MECP2*-null mice [12], which is encouraging as the extrasynaptic GABA receptors (GABA_A_Rs) may provide an alternative pharmaceutical target to relieve the excessive neuronal excitability and its associated RTT symptoms. Indeed, we have found that the extrasynaptic GABA_A_R agonist 4,5,6,7-tetrahydroisoxazolo[5,4-c]pyridin-3-ol hydrochloride (THIP) is beneficial to RTT-like symptom relief in *MECP2* ^−/Y^ mice.

THIP or gaboxadol is an investigational drug, originally developed for insomnia. Clinical trials suggest that THIP (10 mg/day) has no significant effects on sleep onset and total sleep time [13]. It does have effects on these measures in a higher dose (15 mg) where the effects are inconsistent between genders, and side effects emerge including sedation and disorientation [13, 14]. Therefore, Merck and Lundbeck canceled further development of the drug. It is not unusual, however, that a preclinical drug fails in one application but succeeds in another. The low efficacy of THIP on insomnia indeed may be beneficial for its applications to RTT, as the unnecessary sedation can be avoided. We have found that intraperitoneal injection of THIP alleviates the breathing abnormalities and extends lifespans of *MECP2*-null mice [12]. However, intraperitoneal injection may introduce stress and subject the animals to infection. To overcome this potential problem, oral administration was given to mice in this study. Also, we chose to use a low and non-sedative dose of THIP to avoid potential side effects. RTT symptoms start 6–18 months after birth, causing a loss of certain acquired motor and language skills in humans. To intervene in this early period of development, we exposed neonatal mice to THIP 1 day after birth before the RTT-like symptoms manifest themselves. Therefore, this study was conducted in a way that was close to therapeutic condition and very much different from our previous study [12].

## Methods

### Animals

All experimental procedures were conducted in accordance with the National Institutes of Health (NIH) Guide for the Care and Use of Laboratory Animals and were approved by the Georgia State University Institutional Animal Care and Use Committee. Female heterozygous mice (genotype: *MECP2* ^*−/+*^; strain name: B6.129P2(C)-*MECP2* ^tm1.1Bird^/J; stock number 003890) from Jackson Lab were crossbred with male C57BL/6 mice (wild type (WT)) to produce the *MECP2*-null mice with the genotype *MECP2* ^−/Y^. The PCR protocol from Jackson Lab was used to identify the genotype. All experiments were done in male mice because the *MECP2* ^−/Y^ males offer a completely *MECP2*-null condition that is not always available in *MECP2* ^−/+^ females owing to uncontrolled X-chromosome inactivation.

### THIP administration

THIP was given to the mother in her drinking water (200 mg/L) and then passed to pups of WT and *MECP2* ^−/Y^ male mice via lactation [15]. This will last till weaning at P18. After that, THIP was given through the pup’s drinking water (20 mg/L) for another 5 weeks.

### Brain slice preparation

Experiments were performed as we described previously [7]. In brief, mice were decapitated after deep anesthesia with inhalation of saturated isoflurane. The brain stem was obtained and immediately placed in ice-cold and sucrose-rich artificial cerebrospinal fluid (aCSF) containing (in mM) 220 sucrose, 1.9 KCl, 0.5 CaCl_2_, 6 MgCl_2_, 33 NaHCO_3_, 1.2 NaH_2_PO_4_, and 10 D-glucose. The solution was bubbled with 95 % O_2_ balanced with 5 % CO_2_ (pH 7. 40). The transverse pontine sections (150–250 μm) containing the LC area were obtained using a vibratome sectioning system and then recovered at 33 °C for 60 min in normal aCSF containing (in mM) 124 NaCl, 3 KCl, 2 CaCl_2_, 2 MgCl_2_, 26 NaHCO_3_, 1.3 NaH_2_PO_4_, and 10 D-glucose. The brain slices were kept at room temperature before use. During recording, the slices were perfused with oxygenated aCSF at a rate of 2 ml/min and maintained at 34 °C in a recording chamber by a dual automatic temperature control (Warner Instruments).

### Electrophysiology

Whole-cell current clamp was performed on LC neurons in brain slices. Patch pipettes with resistance as 3–5 MΩ were pulled with a Sutter pipette puller (Model P-97, Novato, CA). Only the neurons with membrane potential less than −40 mV (LC neurons) and action potential larger than 65 mV were accepted for further experiments. The pipette solution contained (in mM) 130 K gluconate, 10 KCl, 10 HEPES, 2 Mg-ATP, 0.3 Na-GTP, and 0.4 EGTA (pH 7. 3). The bath solution was normal aCSF bubbled with 95 % O_2_ and 5 % CO_2_ (pH 7. 40). Recorded signals were amplified with an Axopatch 200B amplifier (Molecular Devices, Union City, CA), digitized at 10 kHz, filtered at 2 kHz, and collected with the Clampex 8.2 data acquisition software (Molecular Devices). Paired experiments were done by three people double-blindly.

Membrane potentials were measured without any current injection. The input resistance was calculated as the slope of the linear portion in the *I*-*V* curve in response to a series of injected pulse hyperpolarized currents (typically from 0.15 to 0 nA). The action potential overshoot was measured as the amplitude from 0 mV to the peak of more than 20 events. The threshold was determined at the initiation point of at least 20 spontaneous action potentials.

### Quantitative PCR

Mice 5–7 weeks old were used in the experiments. Transcripts were obtained from the pontine slices containing the LC. Using complementary DNAs (cDNAs) synthesized with the high-capacity cDNA reverse transcription kit (Life Technologies, Grand Island, NY), quantitative PCR (qPCR) was performed with Fast SYBR® Green Master Mix (Applied Biosystems, Life Technologies, New York, NY) in a fast real-time PCR system (Applied Biosystems 7500) for 40 cycles. GAPDH was used as the internal control for the quantification of tyrosine hydroxylase (TH) and dopamine β hydroxylase (DBH) expression. The following are PCR primers for the experiments: GAPDH (forward: CCAGCCTCGTCCCGTAGA; reverse: TGCCGTGAGTGGAGTCATACTG), TH (forward: TGGCTGACCGCACATTTG; reverse: CCTGCACCGTAAGCCTTCA), DBH (forward: TACCACAACCCACGGAAGATA; reverse: CGGTCAACACAAAGGCAGTCT), δ subunit (forward: GGCTTCTTGGGCTTTACC; reverse: CACCCCCACTGTTTTTCTC), α6 subunit (forward: GACTTTGCCCATCGTTCC; reverse: TGCAAAAGCTACTGGAAGAG), β1 subunit (forward: TGGTTTTCGATCTTGTGTGTCAG; reverse: AGCCACCTCTCTCTTTGTGTTTG), and β2 subunit (forward: TTCCCACTGCTGTTTCTCACATAC; reverse: ATCCTAACCACTTCTCCTTTTTTCC).

### Western blot

Pontine tissues containing the LC were obtained from 5- to 7-week-old mice and processed in RIPA buffer (Sigma-Aldrich, St. Louis, MO) with 1 % protease inhibitor. BCA protein assay reagent (Pierce, Rockford, IL) was used to estimate the protein concentrations using 30 μg proteins to detect TH and DBH signals in 10 % SDS-PAGE gels and electrophoretically transferred to nitrocellulose membranes. The membranes were then blocked for 2 h in 5 % non-fat milk and incubated overnight at 4 °C with rabbit GAPDH primary antibodies (1:10,000, Sigma-Aldrich, St. Louis, MO), rabbit TH (1:1000, Sigma-Aldrich, St. Louis, MO), and DBH primary antibodies (1:1000, Sigma-Aldrich, St. Louis, MO). After being washed in PBS twice, the membranes were incubated by horseradish peroxidase (HRP) conjugate goat anti-rabbit secondary antibodies (1:5000, Life Technologies, Frederick, MD) for 1 h in room temperature. The chemiluminescent detection system (Pierce) was used to expose the membrane to films (Hy Blot CL; Denville, Metuchen, NJ), and the photographs were scanned. The immunoblotting signals were quantified using the ImageJ software (NIH). TH and DBH signals were normalized to the internal GAPDH controls.

### Plethysmograph recording

Breathing activity of conscious mice was recorded with the plethysmograph system consisting of a ~40 ml test chamber, a reference chamber, and a force-electricity transducer. The individual animal was kept in the test chamber flowed by air at a rate of 60 ml/min. The mouse was allowed to adapt to the chamber for at least 20 min followed by a 20-min recording. The breathing activity was recorded continuously as the barometrical changes between the test chamber and the reference chamber with the force-electricity transducer. The signal was amplified and then collected with a Pclamp 9 software. The animals were monitored via a video camera to ensure the wake status during tests. The data analysis was done double-blindly to the treatment. Apnea was considered only if the breathing cycle lasts twice longer than the previous one. Breathing frequency variation was calculated as the ratio of standard deviation (SD) over the arithmetic mean of breathing frequency. The SD and arithmetic mean were measured from 200~300 successive breathing events, which were randomly sampled from three or four stretches with at least 50 breaths in each.

### Grip strength

When lifted by the tail, the forelimbs of a mouse (age 5–6 weeks) were allowed to grasp the sensor lever of a force-electricity transducer. The mouse was then gently pulled upward by the tail until it released the grip. Forces were continuously recorded with the Clampex 9 software. The grip strength of each mouse was measured as the maximum force before lever release, and averaged from three consecutive trails.

### Grid walking

A mouse (age 5–6 weeks) was placed on the metal rigid floor of a trial box (32 cm × 20 cm × 20 cm). The box was elevated by 50 cm with the floor made of 11 × 11 mm metal mesh. Mouse walking on the metal mesh floor was videotaped for 5 min. In the video record, the limb placement error was counted. A footfault was counted only when a limb missed the metal floor bar (0.5 mm in diameter) completely and went through the grid opening. The footfault ratio was calculated by the overall number of footfaults divided by the total steps including both forelimbs and hindlimbs.

### Open field test

The experiment was performed as we described previously [16]. Mice aged 5–6 weeks were tested in an open field chamber made of white plexiglass boards (50 cm L × 50 cm W × 30 cm H) with 10 cm × 10 cm square lines. Test animals were kept in their home cages and habituated for 30 min in the test room before testing. When tested, each animal was placed in the center square and allowed to move freely in the chamber. Spontaneous locomotion activity was monitored by a video camera for 5 min. With the video record, square crosses (all four paws cross) were measured in each mouse. To eliminate potential residual odors and potential contaminants, 70 % ethanol was used to clean the apparatus followed by dd H_2_O rinse after each test.

### Social interaction

Mice, age 6–7 weeks, were tested in a box (60 cm L × 30 cm W × 40 cm H), in which there were three chambers (20 cm L × 30 cm W × 40 cm H) separated with transparent walls. A door was arranged diagonally in each wall allowing the tested mouse to travel freely in the chambers. Before test, mice were placed in the test room for 30 min habituation. Then sequential tests were performed in each mouse. Firstly, the tested mouse was placed in the center chamber and allowed to move freely over all three chambers for 10 min. Its chamber preference was analyzed by the time spent in each chamber. Secondly, the social behavior test was performed by introducing a random littermate in one of the side chambers for 10 min, while times that the tested mice spent with the mice were measured. The littermate was randomly assigned in either side of the chamber to avoid the side bias. Lastly, social novelty test was performed by introducing a new stranger mouse in the chamber and switching the familiar littermate to the other chamber. The time spent in both side the chambers were analyzed subsequently [17].

### Lifespan

*MECP2* ^−/Y^ mice used in the experiment were randomly selected and divided into two groups. One group was treated with THIP containing water and the other treated with regular water as vehicle control. Their lifespan were monitored under identical living conditions. Their daily activity and general physical conditions, including feeding, movement, body weight, and interaction with other mice, were observed. Death date of each mouse was recorded when it occurred naturally or reached the humane end point that was determined by staff members in the animal facility at Georgia State University without any consultation with the investigators. One outlier, which was 1.5 interquartile range (IQR) above the third quantile and below the first quantile, was removed from each group to minimize data variations.

### Randomization/double blind

The animals used in the study were randomly separated into a vehicle group and THIP group. The patch experiments were done double-blindly by two to three people without information of mouse genotype and treatment. All the data analysis of behavior experiments, including breathing activity, motor function, and social behavior, were done with no information of the genotype and treatment.

### Data analysis

The sample sizes in the experiments were examined with G-Power Analysis to yield sufficient statistical power. Data are presented as means ± SE or median ± IQR. Mantel-Cox test was used in the lifespan experiment. Kruskal-Wallis test, Pearson correlation, and Spearman’s correlation were used in the breathing experiments. ANOVA and Tukey’s post hoc were applied in the behavior tests and electrophysiology experiments. Student’s *t* test was performed to analyze the data in the molecular experiments. Difference was considered significant when *P* < 0.05.

## Results

### THIP administration

Symptoms of RTT patients and mouse models start after a period of postnatal development. In *MECP2*-null mice, breathing disorders started at 2–3 weeks after birth, and defects in motor and social behaviors begin at 4–6 weeks [18, 19]. Early intervention to extrasynaptic GABA_A_Rs may affect the development of the symptoms. Therefore, we started the THIP treatment of *MECP2*-null mice from the birth day and maintained the level till mice were fully mature.

The following strategies were used to determine THIP dosing. (a) Based on water consumptions in our studies, the dose given to the mother was 61.0 ± 2.2 mg/kg/day. Consistent with previous studies, the mother with this dose did not show any evident sedation neither any behavioral alterations [15]. The infants received maximally one tenth of the dose to the mother via lactation [20–22], i.e., ~6 mg/kg/day. After weaning, these mice received 6.3 ± 0.4 mg/kg/day THIP in their drinking water, which were also calculated based on their daily water intake. (b) According to a THIP patent report, the LD50 in mice is 320 mg/kg orally, which is 2.2 times higher than i.p. (LD50 145 mg/kg) [23]. The THIP dose used in mouse models of Angelman syndrome and Fragile X syndrome is 2–3 mg/kg i.p. [24–26], equivalent to 5–7 mg/kg in oral after multiplication by 2.2, which is approximately the same as used in our studies. (c) THIP pharmacokinetics has been well studied in humans and laboratory animals [27–30]. According to the visual observation, THIP treatment had no evident effects on feeding, movement, body weight, and other general physical conditions in both WT and *MECP2*-null mice.

### Lifespan

**Fig. 1:**
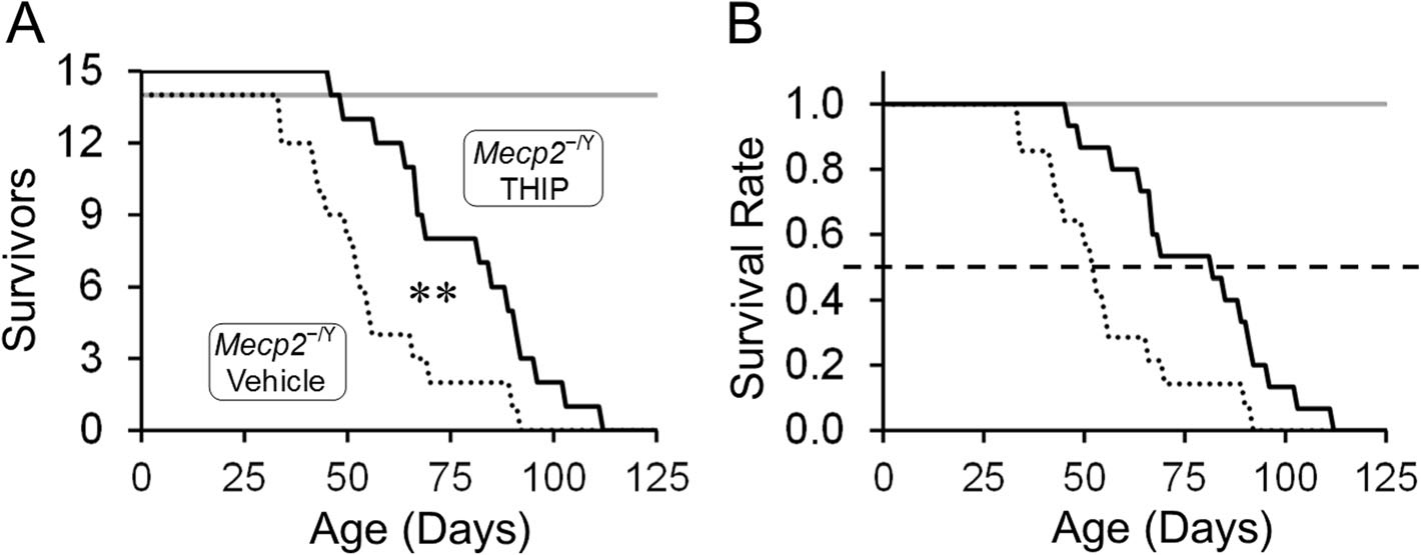
THIP administration extended the lifespan of *MECP2*-null mice. **a** Twenty-nine *MECP2-null* mice were used in the survival experiment and 14 of them were delivered THIP orally (*solid line*) and 13 without THIP treatment (*dashed line*). (**P < 0.01; Mantel-Cox test). **b** Percentage of survival in the tested mice. In the vehicle group, 50 % *MECP2*-null mice died within 52 days, while THIP treatment expanded the 50 % lifespan to 82 days

The lifespan of the mice was studied with THIP or vehicle treatment. In the vehicle group, about 50 % of the *MECP2*-null animals died at P52 with only 1 out of 14 tested animals surviving beyond P80. In contrast, *MECP2*-null mice with THIP treatment reached 50 % fatality (LD50) on P82, and one third (5 out of 15) mice lived beyond P90 (Fig. 1a, b). When comparing LD50, the THIP administration extended the lifespan of *MECP2*-null mice by over 50 %, which was statistically significant as well (Fig. 1a; *P* = 0.004, Mantel-Cox test). The same THIP and vehicle treatments did not cause any lethality in WT mice.

### Breathing abnormalities

**Fig. 2:**
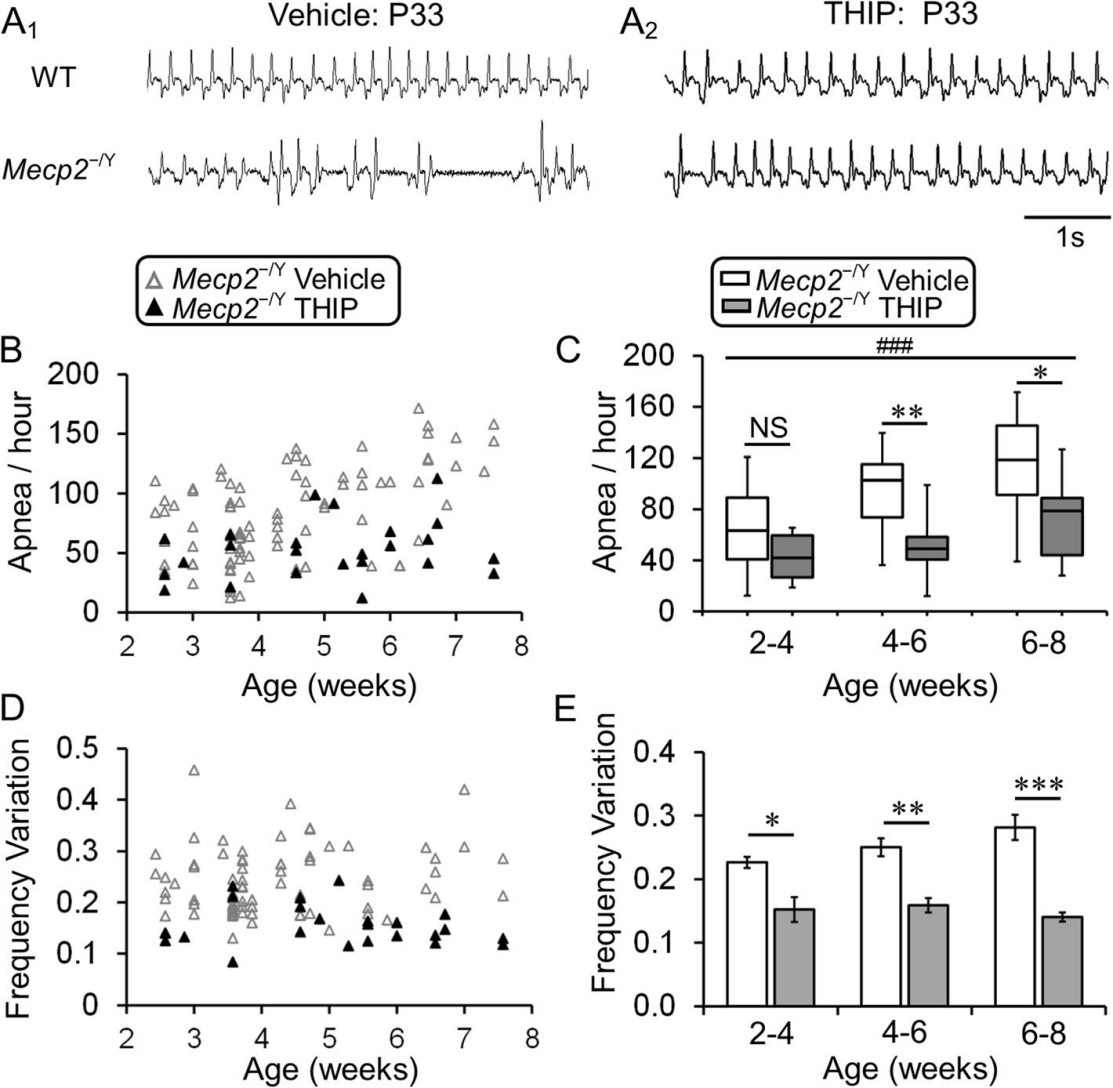
THIP administration alleviated the breathing abnormalities in *MECP2*-null mice. **A**_**1**_, **A**_**2**_ Typical records of breathing activity from both WT and *MECP2*-null mice with and without THIP administration. **B** Distributions of apnea count in different aged *MECP2*-null mice with and without THIP treatment. **C** In *MECP2*-null mice, THIP administration significantly reduced the apnea count at ages of 4–6 weeks (vehicle: *n* = 26, THIP: *n* = 8, *P* = 0.002) and 6–8 weeks (vehicle: *n* = 19, THIP: *n* = 8, *P* = 0.021), although the significance was not found in 2–4 weeks (vehicle: *n* = 45, THIP: *n* = 7, *P* = 0.081; ^###^ *P* < 0.001 in Kruskal-Wallis test; *P < 0.05, **P < 0.01 in Mann-Whitney post hoc comparison). **D**, **E** Similar effects of THIP treatment on breathing frequency variation was observed in these mice (2–4 weeks: *P* = 0.037; 4–6 weeks: *P* = 0.004; 6–8 weeks: *P* < 0.001; *P < 0.05, **P < 0.01, ***P < 0.001; one-way ANOVA and Tukey’s post hoc)

Like people with RTT, *MECP2*-null mice developed severe breathing abnormalities showing significantly high apnea rate and high breathing frequency variation [6, 31], which may lead to the early death or unexpected sudden death seen in RTT patients and the RTT mouse model. It is possible that the extended lifespan in *MECP2*-null mice is attributable to the alleviation of breathing abnormalities through THIP treatment. Therefore, we studied mouse breathing activity using plethysmography. Early-life administration of THIP prevented the development of breathing abnormalities in *MECP2*-null mice (Fig. 2B, D). In age 6–8 weeks, both the apnea rate and breathing frequency variation were significantly reduced in *MECP2*-null mice treated with THIP (Fig. 2A_1_, A_2_, C, E).

### Motor function

**Fig. 3:**
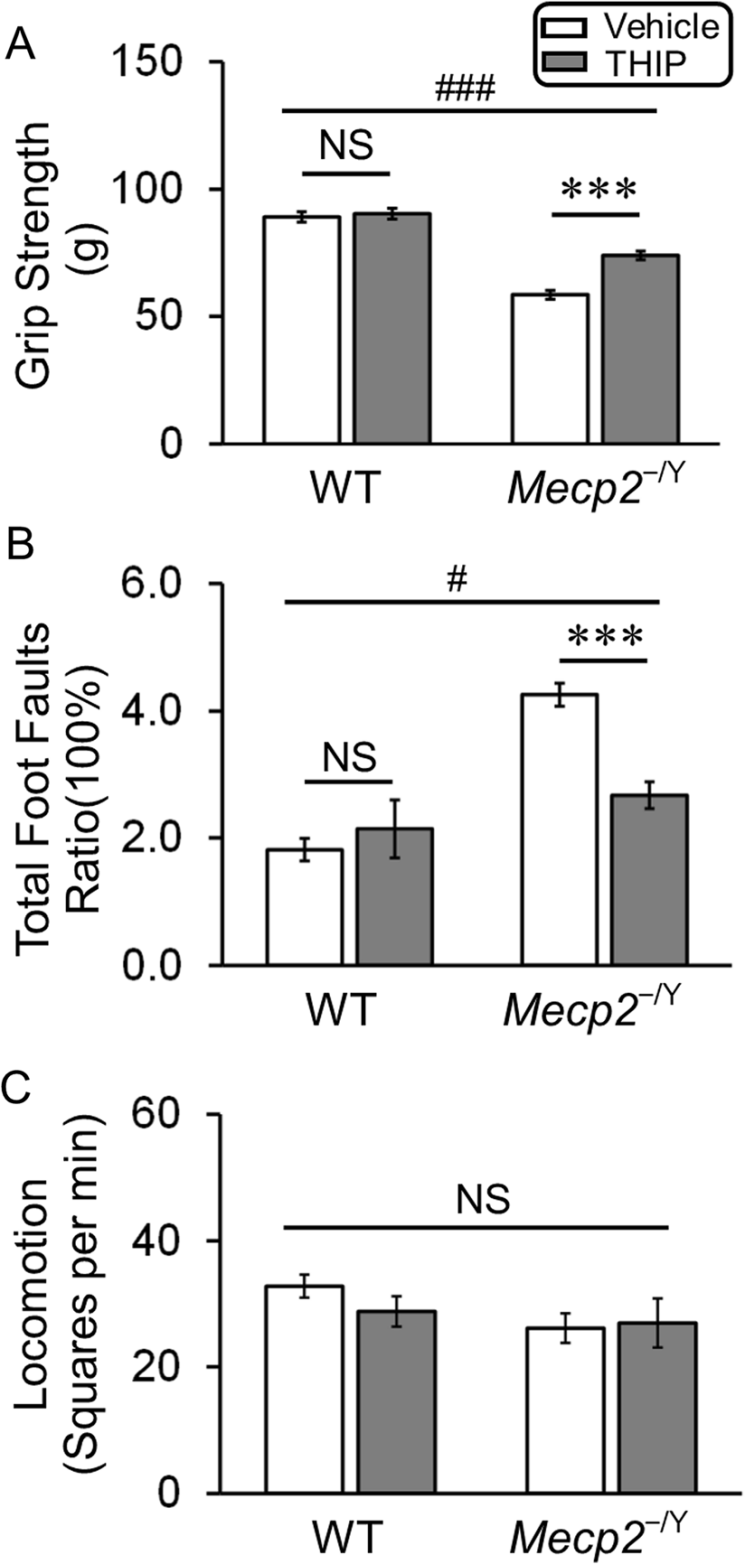
THIP administration improved motor function of *MECP2*-null mice. **a** Significant main effects of THIP treatment (*F* = 23.74, *df* = 1, *P* < 0.001) and genotype (*F* = 147.85, *df* = 1, *P* < 0.001) were observed, as well as a significant interaction (*F* = 12.04, *df* = 1, *P* < 0.001). ^(###^ *P* < 0.001, two-way ANOVA). The grip strength of *MECP2*-null mice was significantly increased with THIP treatment (WT: *n* = 18 and *n* = 18 mice; *MECP2*-null: *n* = 23 and *n* = 22; vehicle and THIP, respectively; ***P < 0.001, Tukey’s post hoc). **b** Significant main effects of THIP treatment (*F* = 5.26, *df* = 1, *P* < 0.05) and genotype (*F* = 30.4, *df* = 1, *P* < 0.001) were observed, as well as a significant interaction (*F* = 13.25, *df* = 1, *P* < 0.001) (^#^ *P* < 0.05, two-way ANOVA). THIP administration significantly reduced the footfault ratio (including both hindlimb and forelimb) of *MECP2*-null mice (WT: *n* = 22 and *n* = 23 mice; *MECP2*-null: *n* = 20 and *n* = 25; vehicle and THIP, respectively; ***P < 0.001, Tukey’s post hoc). **c** The spontaneous locomotion of WT and *MECP2*-null mice was not significantly affected by THIP treatment. The main effect of THIP treatment was not significant (*F* = 0.26, *df* = 1, *P* = 0.614), as the main effect of genotype (*F* = 3.00, *df* = 1, *P* = 0.095). The interaction of these two factors was not significant (*F* = 0.99, *df* = 1, *P* = 0.329) (WT: *n* = 8 and *n* = 7 mice; *MECP2*-null: *n* = 9 and *n* = 6; vehicle and THIP, respectively; two-way ANOVA)

The grip strength and grid walking tests were performed to evaluate muscle strength and motor coordination, respectively. Our results showed that in 5- to 6-week-old *MECP2*-null mice, THIP treatment improved the grip strength from 58.5 ± 2.1 g to 74.0 ± 1.7 g (Fig. 3a) and the footfault ratio from 4.3 ± 0.5 % to 2.7 ± 0.2 % (Fig. 3b). Both were significantly different from those of the vehicle controls. These results were unlikely to be due to the sedative effects of THIP, as in the open field test THIP did not affect the spontaneous locomotion of either WT or *MECP2*-null mice (Fig. 3c). Therefore, chronic treatment of THIP moderated certain motor defects in *MECP2*-null mice, such as muscle strength and motor coordination.

### Social behaviors

**Fig. 4:**
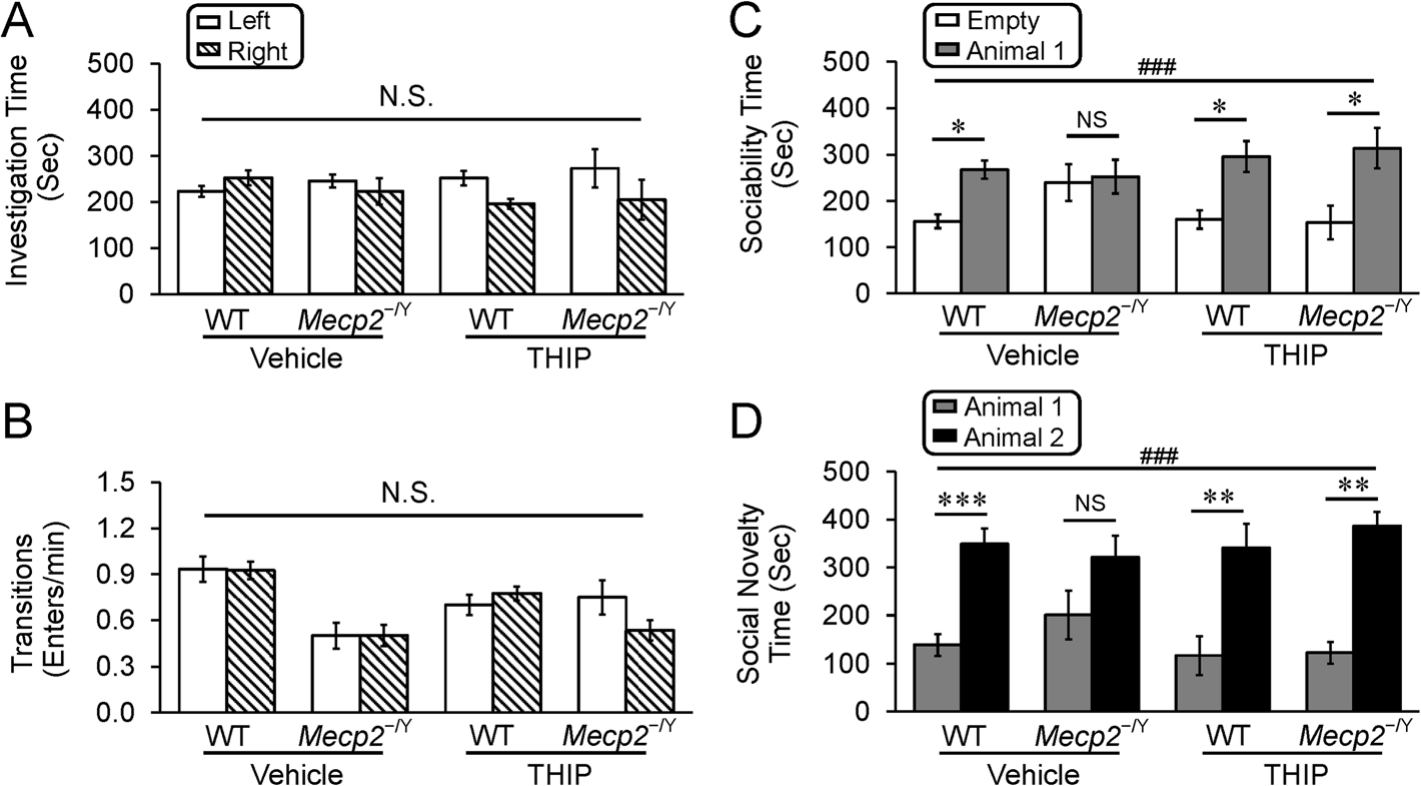
THIP administration alleviated the defects of social behaviors in *MECP2*-null mice. During the habituation period in the three chamber test, both WT and null mice took similar amount of times (**a**) and transitions (**b**) in either side of the chambers indicating no preference. The main effect preference was not significant. (**a** *F* = 1.72, *df* = 1, *P* = 0.195; **b** *F* = 0.20, *df* = 1, *P* = 0.656; three-way ANOVA). The transitions between the chambers also suggested that the tested animals are not in a sedative state. **c** In the sociability test, a significant difference was detected within the main factor of preference (*F* = 23.31, *df* = 1, ^###^ *P* < 0.001, three-way ANOVA). WT mice spent significantly more time in the chamber containing an animal than the empty one, whereas the *MECP2*-null mice lost such a preference. THIP administration increased the time expenditure of *MECP2*-null mice in interacting with another mouse (*P < 0.05; Tukey’s post hoc). No significant differences were found in the main factor of genotype (*F* = 1.20, *df* = 1, *P* = 0.278) or THIP treatment (*F* = 0.03, *df* = 1, *P* = 0.863). The interactions of genotype × treatment (*F* = 0.46, *df* = 1, *P* = 0.500), genotype × preference (*F* = 1.41, *df* = 1, *P* = 0.239), treatment × preference (*F* = 3.31, *df* = 1, *P* = 0.074), or genotype × THIP treatment × preference (*F* = 2.08, *df* = 1, *P* = 0.155) were not significant as well (three-way ANOVA). **d** In the social novelty test, the main factor of preference showed a significant difference (*F* = 54.48, *df* = 1, ^###^ *P* < 0.001, three-way ANOVA). WT mice spent significantly more time in the chamber with a novel animal than the chamber with a familiar one, whereas the *MECP2*-null mice did not show the preference to either chamber. With THIP treatment the novelty preference was improved in the *MECP2*-null mice (**P < 0.01, ***P < 0.001; Tukey’s post hoc). No significant differences were found in the main factor of genotype (*F* = 0.57, *df* = 1, *P* = 0.453) or THIP treatment (*F* = 0.17, *df* = 1, *P* = 0.681). The interactions of genotype × treatment (*F* = 0.02, *df* = 1, *P* = 0.888), genotype × preference (*F* = 0.49, *df* = 1, *P* = 0.487), treatment × preference (*F* = 1.58, *df* = 1, *P* = 0.213), or genotype × THIP treatment × preference (*F* = 1.35, *df* = 1, *P* = 0.250) were not significant as well (vehicle: *n* = 12 and *n* = 9; THIP: *n* = 8 and *n* = 6; WT and *MECP2*-null, respectively; three-way ANOVA)

The three-chambered tests are used widely in the studies of sociability and social novelty [17]. In our current study, only the animals showing no preference to either side chamber during the exploration period were used for further testing (Fig. 4a). Both of the WT and *MECP2*-null mice in the experiments showed similar time spending in the side chambers, indicating that none of the animals were in the sedative state. THIP treatment did not alter the chamber transitions or the chamber preference either (Fig. 4b).

In the sociability test, we found that WT mice tended to spend significantly longer time in the chamber with an animal than without (267.9 ± 16.7 s in the animal chamber vs. 155.9 ± 14.6 s in the empty chamber), whereas *MECP2*-null mice in the vehicle group did not show such preference (252.7 ± 36.3 s in the animal chamber 1 vs. 240.0 ± 39.4 s in the empty). THIP administration improved significantly the social interaction or sociability of the *MECP2*-null mice (313.5 ± 43.1 s in the animal chamber 1 vs. 153.5 ± 35.9 s in the empty) (Fig. 4c).

In the social novelty preference test, WT mice spent significantly more time in the chamber with novel animals than the chamber with the familiar one (349.3 ± 32.1 s in the animal 1 chamber vs. 138.4 ± 22.8 s in the animal 2 chamber), whereas the *MECP2*-null mice did not show such a preference (321.7 ± 44.1 s in the animal 1 chamber vs. 200.9 ± 50.9 s in the animal 2 chamber). THIP treatment improved significantly the social novelty preference of the *MECP2*-null mice (386.7 ± 29.1 s in the animal 1 chamber vs. 122.2 ± 22.6 s in the animal 2 chamber) (Fig. 4d), suggesting that THIP treatment seems to alleviate the defects of sociability and social novelty as well.

### Neuronal hyperexcitability

One of the common features of RTT in humans and animal models is the defect in the NE system [19, 32, 33]. Previous studies have shown that excitability of LC neurons increases in *MECP2*-null mice [4, 7]. The LC neurons are the main source of NE in the CNS, and play an important role in breathing regulation, locomotion, arousal, emotion, and other behaviors [3, 6, 34]. Therefore, we chose these neurons to find whether THIP treatment may stabilize their excitability in *MECP2*-null mice.

**Fig. 5:**
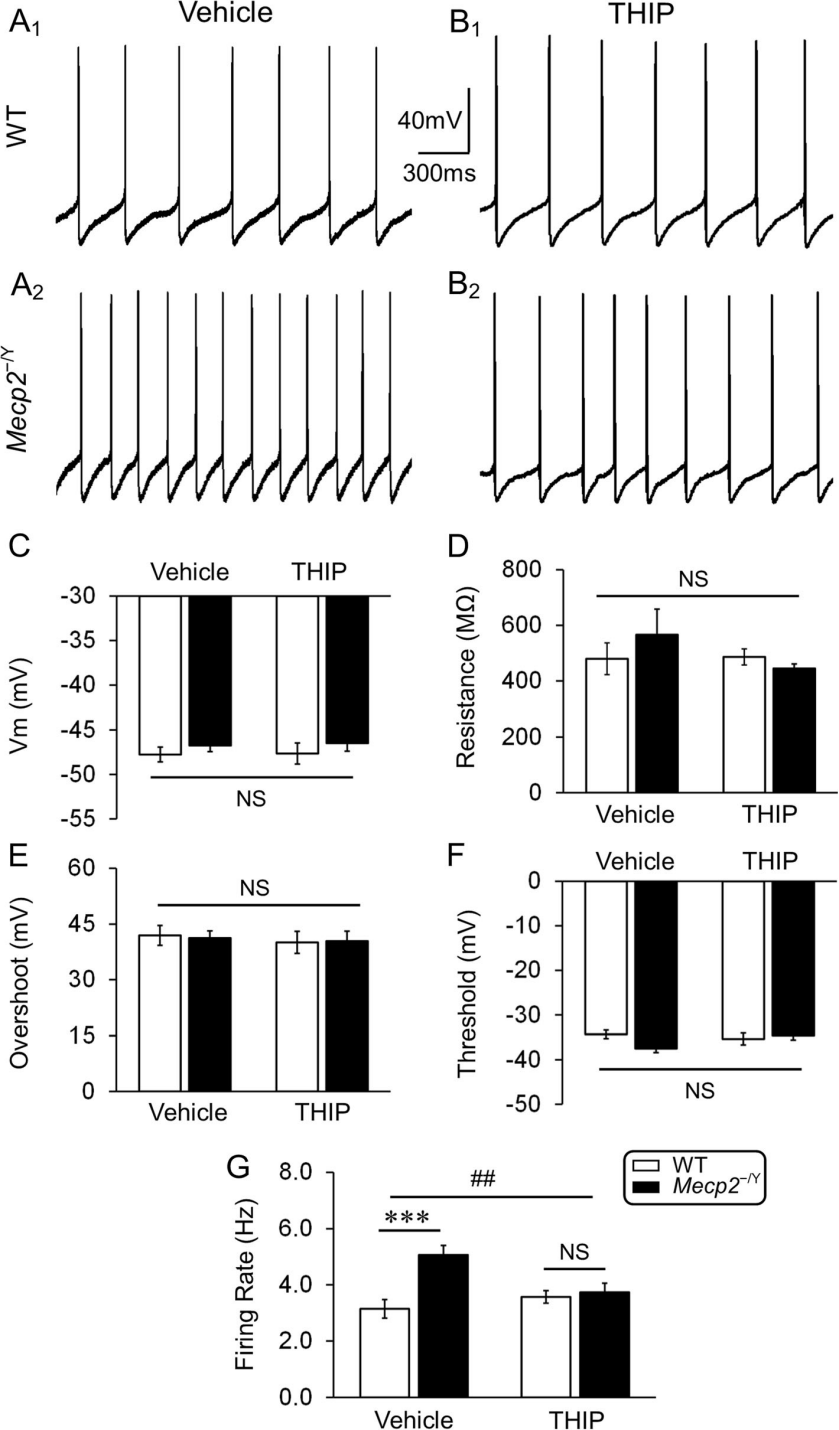
THIP administration suppressed the hyperexcitability of LC neurons in *MECP2*-null mice. **A**_**1**_, **A**_**2**_ Typical recordings of spontaneous firing of LC neurons in WT and *MECP2*-null mice at 1 month of age without THIP treatment. **B**_**1**_, **B**_**2**_ Spontaneous firing of LC neurons in WT and *MECP2*-null mice of the same age with THIP pretreatment. **C**–**F** THIP administration did not significantly change membrane potentials, input resistance, action potential overshoot, and action potential threshold in both WT and *MECP2*-null mice. No significant main effect of THIP treatment (*F* = 0.09, *df* = 1, *P* = 0.765; *F* = 1.15, *df* = 1, *P* = 0.289; *F* = 0.27, *df* = 1, *P* = 0.606; *F* = 0.76, *df* = 1, *P* = 0.387; **C**, **D**, **E**, **F**, respectively) and genotype (*F* = 1.45, *df* = 1, *P* = 0.234; *F* = 0.09, *df* = 1, *P* = 0.765; *F* = 0.01, *df* = 1, *P* = 0.921; *F* = 0.99, *df* = 1, *P* = 0.324; **C**, **D**, **E**, **F**, respectively) were observed, either the interaction (*F* = 0, *df* = 1, *P* = 1.000; *F* = 1.46, *df* = 1, *P* = 0.232; *F* = 0.03, *df* = 1, *P* = 0.863; *F* = 3.58, *df* = 1, *P* = 0.064; **C**, **D**, **E**, **F**, respectively). **G** The main effect of genotype was significant (*F* = 10.06, *df* = 1, *P* < 0.01), whereas the main effect of THIP treatment was not (*F* = 1.72, *df* = 1, *P* = 0.196). The interaction of these two factors was significant (*F* = 8.6, *df* = 1, *P* < 0.01) as well (^##^ *P* < 0.01; two-way ANOVA). The firing activity of LC neurons in *MECP2*-null mice is significantly increased compared to the WT and chronic treatment with THIP abolished the hyperexcitability (vehicle: *n* = 14 and *n* = 13; THIP: *n* = 13 and *n* = 16; in WT and *MECP2*-null, respectively; ***P < 0.001; Tukey’s post hoc)

In the brain slice preparation, whole-cell current clamp was performed in LC neurons from mice with and without THIP pretreatment. Of four groups of mice, only the LC neurons from *MECP2*-null mice in vehicle control showed an obvious increase in spontaneous firing activity (Fig. 5A_1_, A_2_, B_1_, B_2_). Detailed analysis of the passive and active membrane properties showed that the THIP pretreatment did not significantly change membrane potential, input resistance, action potential overshoot, and firing threshold of either groups of neurons (Fig. 5C, D, E, F). In *MECP2*-null neurons, the spontaneous firing rate was significantly higher than that in WT. The THIP pretreatment, however, abolished the difference (vehicle: 3.1 ± 0.3 Hz and 5.1 ± 0.3 Hz; THIP: 3.6 ± 0.2 Hz and 3.7 ± 0.3 Hz; WT and *MECP2*-null, respectively; Fig. 5G). Thus, these results suggest that the LC neuronal hyperexcitability in *MECP2*-null mice is significantly reduced after THIP exposure.

### Gene expressions

**Fig. 6:**
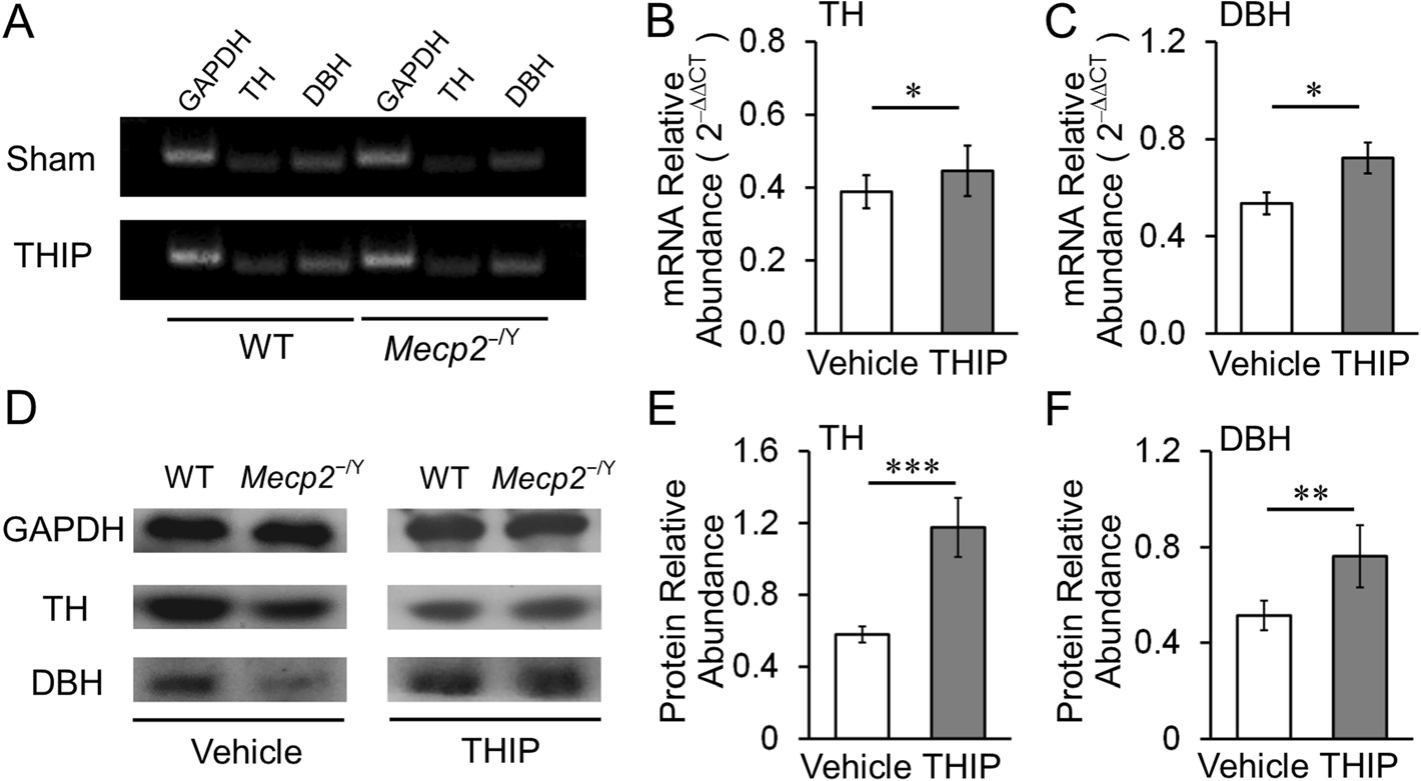
Improvement of TH and DBH expressions with THIP administration in *MECP2*-null mice. **a**-**c**, qPCR analysis showed that during THIP treatment (P37), both TH and DBH transcript levels were significantly increased (vehicle: *n* = 4 and *n* = 4 animals; THIP: *n* = 5 and *n* = 5 animals; WT and *MECP2*-null, respectively). **d**-**f**, The Western analysis also indicated that THIP treatment significantly increased the protein expressions of both TH and DBH (vehicle: *n* = 4 and *n* = 4 animals; THIP: *n* = 4 and *n* = 4 animals; WT and *MECP2*-null, respectively; *P < 0.05, **P < 0.01, ***P < 0.001; one-tailed Student’s *t* test)

Previous studies have shown that the *MECP2* disruption leads to reductions in NE content in the CNS and expression levels of the rate-limiting enzymes TH and DBH for NE biosynthesis [4, 19, 35, 36]. Persistent hyperexcitation of LC neurons may interfere with the homeostatic state in NE biosynthesis and release, leading to the reduced expression of TH and DBH in *MECP2*-null mice [12]. Thus, moderation of LC neuronal hyperexcitability may improve expression of TH and DBH in *MECP2*-null mice. To test this possibility, we studied the TH and DBH at mRNA and protein levels. The qPCR analysis showed that THIP treatment significantly increased both TH and DBH transcript levels in the pontine extracts of *MECP2*-null mice (Fig. 6a–c). Western blot analysis showed a ~2-fold increase in TH protein level and a ~1.5-fold increase in DBH protein level (Fig. 6d–f).

**Fig. 7:**
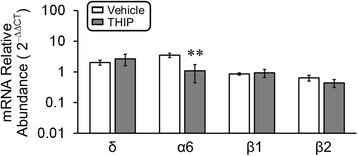
Alteration of GABA_A_R subunits in the LC area of *MECP2*-null mice. qPCR analysis indicated that the mRNA levels of δ and α6 subunits were 2.0 and 3.5 times higher than the WT levels, while THIP treatment significantly reduced the expression level of α6 subunit, without alteration of δ, β1, and β2 subunits (vehicle: *n* = 4 and *n* = 4 animals; THIP: *n* = 5 and *n* = 5 animals; WT and *MECP2*-null, respectively; **P < 0.01; Student’s *t* test)

Early-life exposure to THIP might also reshuffle the GABA receptor subunits. Therefore, quantitative PCR was performed to detect the mRNA levels of δ, α6, β1, and β2 subunits, which were reported as significantly changed in *MECP2*-null LC area. In comparison with vehicle control, a significant reduction of α6 subunit was detected in *MECP2*-null mice with THIP treatment, while no significant changes in other subunit expression were found (Fig. 7).

## Discussion

In these studies, we have shown that early-life exposure of the *MECP2*-null mice to a non-sedative dose of the extrasynaptic GABA_A_Rs agonist THIP has several beneficial effects on lifespan, breathing activity, motor function, and social behaviors. Such alleviation of RTT-like symptoms by THIP treatment is associated with the stabilization of neuronal excitability and enhancement of NE biosynthetic enzymes in LC neurons.

The extrasynaptic GABA_A_Rs have several properties different from the synaptic GABA_A_Rs, which may be unique in interventions to neuronal excitability. They are located outside the synaptic area, produce tonic or long-lasting Cl^−^ currents, show very little desensitization upon activation, and are sensitive to some synaptic GABA_A_R agonists and extrasynaptic GABA_A_R-specific agonists [37–39]. They have the capability to change dynamically their expression levels under different physiological and pathophysiological conditions [12, 40, 41]. Manipulations of these receptors with selective agents do not interrupt GABAergic synaptic transmission mediated by the synaptic GABA_A_Rs. Thus, therapeutic activation of these extrasynaptic GABA_A_Rs may avoid several side effects of the synaptic GABA_A_R activators including sedation, tolerance, and addiction.

People with RTT and the mouse models show the delayed onset and progressive symptoms. Although the mechanism for the delayed symptom onset remains unclear, some factors may contribute to it, such as dynamic spatiotemporal relationship between *MECP2* and methylated DNA [42] and the altered allopregananallone modulation of the GABA system during perinatal period [8]. Thus, intervention to neuronal hyperexcitability before the symptom onset may be a potential way to prevent or delay the development of the disease. The δ subunit containing extrasynaptic GABA_A_Rs are expressed dynamically with growth [41]. Since severe defects in the synaptic GABA_A_R system have been demonstrated in mature *MECP2*-null mice, treatment with THIP in early lives may be beneficial with respect to enforcement of the inhibitory system in the neurodevelopment of *MECP2*-null mice.

The reduced expression of α6 subunits suggests that early treatment of THIP may lower the GABAR expression and the consequent GABAergic inhibition. The α6 subunit is known to contribute to both the extrasynaptic receptors together with the δ subunit and the synaptic GABA_A_Rs with β and γ subunits [43, 44]. The reduction in these GABA_A_Rs might possibly lead to rebound excitation after THIP withdrawal. The stabilization of neuronal excitability, however, might affect cellular mechanisms reducing abnormalities, which could result in a long-term reduction in neuronal hyperexcitabiltiy after THIP withdrawal, contributing to the outcome of THIP in breathing, motor function, social behaviors, and lifespan.

To minimize potential side effects of THIP, we chose to use THIP chronically in low dosage. Although pharmacokinetic studies were not performed in this report, such information has been collected in previous studies on mice, rats, dogs, and humans [27–30]. With a daily dose of 10 mg in humans, THIP reaches the maximum plasma concentration ~140 ng/ml in 2.0 h, and the terminal plasma half-life time is 1.7 h [27]. Another preclinical study in humans, rats, and mice shows a rapid and complete absorption of THIP with the peak concentration reached within 0.5 h in several organs include the brain [29]. A clinical report indicates that therapeutic dosages of THIP by long-term oral administration range from 20 to 120 mg daily in human patients [45]. Higher doses of THIP may cause adverse side effects, including sedation, confusion, and dizziness [14]. In our present study, the oral dose of THIP was calculated to be ~6 mg, which appears effective for alleviating multiple RTT-like symptoms in *MECP2*-null mice. Our test of spontaneous locomotion supports the non-sedative effects of the dosage. The dosage given to the mother during lactation was 61.0 ± 2.2 mg/kg/day, which was also reported to have no sedative effects neither behavioral alterations [15].

Similar to the dosages that we used in the study, several previous studies have reported to use THIP for treatment of mouse models of Fragile X syndrome and Angelman syndrome. Since these diseases share multiple similarities to RTT, such as impaired GABA system, neuronal hyperexcitability, and autism-like symptoms [24, 26, 46], the information shown in the present study is likely to benefit to moving the drug for further clinical trials in all these diseases.

LC neurons, the major NE source in the CNS, project broadly to the other brain regions, including the medulla where the respiratory center is located and the prefrontal cortex where the NE system affects cognitive functions, seizure, and social behaviors [47, 48]. In *MECP2*-null mice with THIP treatment, the target regions of LC-NE projection may benefit from the enhanced NE synthesis, leading to the alleviation of the associated abnormal behaviors. On the other hand, impaired neuron networks were widely seen in *MECP2*-null mice [40, 49]. The social defects of autism spectrum disorders were believed to be correlated to the weak connections in the default networks including the medial prefrontal cortex and posterior cingulate cortex [50]. With the beneficial effects found in this study, we speculate that THIP might contribute to reinforcement of these connections as well as amelioration of the phenotypes.

In comparison to the *MECP2*-null mice, the most widely used RTT mouse model, the *MECP2* ^−/+^ mice tend to display large variations in RTT-like symptoms due to the random X-chromosome inactivation. According to our previous study, only 15–20 % *MECP2* ^−/+^ mice in age 1-6 months showed the RTT-like symptom of breathing abnormalities, suggesting that the wild-type allele is not randomly inactivated [2]. Generally, ~50 % the neurons in the CNS of *MECP2* ^−/+^ mice retained *MECP2* expression [51, 52], which may allow the *MECP2* ^−/+^ mice to recapitulate the normal behaviors to some degree, compared to the *MECP2-*null mice. However, the *MECP2* mosaic expression pattern is not uniform in the CNS and it varies between individuals, ages, and brain regions [51, 52]. Regional expression levels of *MECP2* was reported be correlated to the specific symptoms in the *MECP2* ^−/+^ mice. Hippocampus *MECP2* expression is related to the exploratory activity behaviors and anxiety-like behaviors. Cortical *MECP2* expression affects the general symptomatic severity [53]. The age-dependent mosaic pattern suggests that even the X-chromosome inactivation ratio may also be affected by *MECP2* deficiency in the RTT mice brain and the consequent variation of postnatal brain functions in RTT [51]. Thus, the age-dependent and region-specific expression pattern of *MECP2* in the CNS contributes to the large variation of the phenotypic outcome in *MECP2* ^−/+^ mice, which all need to be considered in studies of female RTT models. Furthermore, *MECP2* ^−/+^ mice usually develop the diagnostic symptoms when they become sexually mature. The periodical hormone alternations in *MECP2* ^−/+^ mice may affect mouse performance in the behavioral tests complicating the interpretation of the THIP effects. A previous study reports significant variations in the open field, tail flick, and suspension tests in female mice during their estrous cycle [54]. With all these complications in the female models, therefore, studies under *MECP2*-null condition in the male model seems beneficial as the first step of investigation before sophisticated preclinical trials are conducted, which can be based on *MECP2* ^−/+^ female mice and may benefit from the experimental evidence obtained from the male RTT models.

Selective restoration of *MECP2* in GABAergic neurons rescues multiple phenotypes in both *MECP2* ^−/Y^ and *MECP2* ^−/+^ mice [55], which suggests that the GABA system is a feasible target to manipulate in RTT female mouse model and RTT patients. Although the sexual difference of LC neurons might be a concern of the potential effects of THIP, a morphological study suggested female LC neurons showed a higher frequency of communication with peri-LC neurons in comparison to the male [56, 57], indicating that THIP may have a greater effect in female RTT mouse model or patients. A previous study reports that for some unknown reasons, THIP tends to have a greater efficacy in women than in men [13], further suggesting the potential beneficial effects of THIP in RTT female mouse model and patients. Nevertheless, further studies on *MECP2* ^−/+^ mice are needed, which may be conducted as deliberate, thorough, and systematic investigations that might benefit from our findings in the male model.

## Conclusions

Consistent with our previous study showing that the daily injection of THIP in a high dose alleviates breathing abnormalities by stabilizing the neuronal activity [12], our current study shows that early-life exposure to a low dose of THIP affects multiple RTT-like symptoms. The early-life exposure to THIP extends the lifespan of *MECP2*-null mice, reduces breathing disorders and motor dysfunction, and improves social behaviors. These beneficial effects are associated with suppression of hyperexcitability and improvement of biosynthesis enzyme expression in *MECP2*-null LC neurons. These results suggest that THIP has beneficial effects on RTT-like symptoms in the mouse model with complete knockout of the *MECP2* gene.

## Abbreviations

aCSF: Artificial cerebrospinal fluid
Gaboxadol or THIP: 4,5,6,7-Tetrahydroisoxazolo[5,4-*c*]pyridin-3-ol hydrochloride
LC: Locus coeruleus
MECP2: Methyl CpG binding protein 2
RTT: Rett syndrome
WT: Wild type

## References

[1] Chahrour M, Zoghbi HY (2007). The story of Rett syndrome: from clinic to neurobiology. Neuron.

[2] Johnson CM, Cui N, Zhong W, Oginsky MF, Jiang C (2015). Breathing abnormalities in a female mouse model of Rett syndrome. J Physiol Sci.

[3] Lioy DT, Wu WW, Bissonnette JM (2011). Autonomic dysfunction with mutations in the gene that encodes methyl-CpG-binding protein 2: insights into Rett syndrome. Auton Neurosci.

[4] Taneja P, Ogier M, Brooks-Harris G, Schmid DA, Katz DM, Nelson SB (2009). Pathophysiology of locus ceruleus neurons in a mouse model of Rett syndrome. J Neurosci.

[5] Ramirez JM, Ward CS, Neul JL (2013). Breathing challenges in Rett syndrome: lessons learned from humans and animal models. Respir Physiol Neurobiol.

[6] Zhang X, Su J, Cui N, Gai H, Wu Z, Jiang C (2011). The disruption of central CO2 chemosensitivity in a mouse model of Rett syndrome. Am J Physiol Cell Physiol.

[7] Zhang X, Cui N, Wu Z, Su J, Tadepalli JS, Sekizar S, Jiang C (2010). Intrinsic membrane properties of locus coeruleus neurons in *MECP2*-null mice. Am J Physiol Cell Physiol.

[8] Jin X, Zhong W, Jiang C (2013). Time-dependent modulation of GABA(A)-ergic synaptic transmission by allopregnanolone in locus coeruleus neurons of *MECP2*-null mice. Am J Physiol Cell Physiol.

[9] Jin X, Cui N, Zhong W, Jin XT, Jiang C (2013). GABAergic synaptic inputs of locus coeruleus neurons in wild-type and *MECP2*-null mice. Am J Physiol Cell Physiol.

[10] Oginsky MF, Cui N, Zhong W, Johnson CM, Jiang C (2014). Alterations in the cholinergic system of brain stem neurons in a mouse model of Rett syndrome. Am J Physiol Cell Physiol.

[11] Medrihan L, Tantalaki E, Aramuni G, Sargsyan V, Dudanova I, Missler M, Zhang W (2008). Early defects of GABAergic synapses in the brain stem of a *MECP2* mouse model of Rett syndrome. J Neurophysiol.

[12] Zhong W, Cui N, Jin X, Oginsky MF, Wu Y, Zhang S, Bondy B, Johnson CM, Jiang C (2015). Methyl CpG binding protein 2 gene disruption augments tonic currents of gamma-aminobutyric acid receptors in locus coeruleus neurons: impact on neuronal excitability and breathing. J Biol Chem.

[13] Roth T, Lines C, Vandormael K, Ceesay P, Anderson D, Snavely D (2010). Effect of gaboxadol on patient-reported measures of sleep and waking function in patients with Primary Insomnia: results from two randomized, controlled, 3-month studies. J Clin Sleep Med.

[14] Kjaer M, Nielsen H (1983). The analgesic effect of the GABA-agonist THIP in patients with chronic pain of malignant origin. A phase-1-2 study. Br J Clin Pharmacol.

[15] Maguire J, Mody I (2008). GABA(A)R plasticity during pregnancy: relevance to postpartum depression. Neuron.

[16] Wu Y, Zhong W, Cui N, Johnson CM, Xing H, Zhang S, Jiang C (2016). Characterization of Rett syndrome-like phenotypes in *MECP2*-knockout rats. J Neurodev Disord.

[17] Samaco RC, Mandel-Brehm C, McGraw CM, Shaw CA, McGill BE, Zoghbi HY (2012). Crh and Oprm1 mediate anxiety-related behavior and social approach in a mouse model of *MECP2* duplication syndrome. Nat Genet.

[18] Kerr B, Alvarez-Saavedra M, Saez MA, Saona A, Young JI (2008). Defective body-weight regulation, motor control and abnormal social interactions in *MECP2* hypomorphic mice. Hum Mol Genet.

[19] Viemari JC, Roux JC, Tryba AK, Saywell V, Burnet H, Pena F, Zanella S, Bevengut M, Barthelemy-Requin M, Herzing LB (2005). *MECP2* deficiency disrupts norepinephrine and respiratory systems in mice. J Neurosci.

[20] Wesson DR, Camber S, Harkey M, Smith DE (1985). Diazepam and desmethyldiazepam in breast milk. J Psychoactive Drugs.

[21] Dusci LJ, Good SM, Hall RW, Ilett KF (1990). Excretion of diazepam and its metabolites in human milk during withdrawal from combination high dose diazepam and oxazepam. Br J Clin Pharmacol.

[22] Borgatta L, Jenny RW, Gruss L, Ong C, Barad D (1997). Clinical significance of methohexital, meperidine, and diazepam in breast milk. J Clin Pharmacol.

[23] Gaboxadol hydrobromide [http://chem.sis.nlm.nih.gov/chemidplus/rn/65202-63-3]. Accessed 8 Oct 2016.

[24] Egawa K, Kitagawa K, Inoue K, Takayama M, Takayama C, Saitoh S, Kishino T, Kitagawa M, Fukuda A (2012). Decreased tonic inhibition in cerebellar granule cells causes motor dysfunction in a mouse model of Angelman syndrome. Sci Transl Med.

[25] Olmos-Serrano JL, Corbin JG, Burns MP (2011). The GABA(A) receptor agonist THIP ameliorates specific behavioral deficits in the mouse model of fragile X syndrome. Dev Neurosci.

[26] Olmos-Serrano JL, Paluszkiewicz SM, Martin BS, Kaufmann WE, Corbin JG, Huntsman MM (2010). Defective GABAergic neurotransmission and pharmacological rescue of neuronal hyperexcitability in the amygdala in a mouse model of fragile X syndrome. J Neurosci.

[27] Boyle J, Danjou P, Alexander R, Calder N, Gargano C, Agrawal N, Fu I, McCrea JB, Murphy MG (2009). Tolerability, pharmacokinetics and night-time effects on postural sway and critical flicker fusion of gaboxadol and zolpidem in elderly subjects. Br J Clin Pharmacol.

[28] Kesisoglou F, Balakrishnan A, Manser K. Utility of PBPK absorption modeling to guide modified release formulation development of gaboxadol, a highly soluble compound with region-dependent absorption. J Pharm Sci. 2016;105:722-28. 10.1002/jps.24674 26457884

[29] Cremers T, Ebert B (2007). Plasma and CNS concentrations of Gaboxadol in rats following subcutaneous administration. Eur J Pharmacol.

[30] Schultz B, Aaes-Jorgensen T, Bogeso KP, Jorgensen A (1981). Preliminary studies on the absorption, distribution, metabolism, and excretion of THIP in animal and man using 14C-labelled compound. Acta Pharmacol Toxicol (Copenh).

[31] Katz DM, Dutschmann M, Ramirez JM, Hilaire G (2009). Breathing disorders in Rett syndrome: progressive neurochemical dysfunction in the respiratory network after birth. Respir Physiol Neurobiol.

[32] Zoghbi HY, Percy AK, Glaze DG, Butler IJ, Riccardi VM (1985). Reduction of biogenic amine levels in the Rett syndrome. N Engl J Med.

[33] Roux JC, Dura E, Moncla A, Mancini J, Villard L (2007). Treatment with desipramine improves breathing and survival in a mouse model for Rett syndrome. Eur J Neurosci.

[34] Chandler DJ (1641). Evidence for a specialized role of the locus coeruleus noradrenergic system in cortical circuitries and behavioral operations. Brain Res.

[35] Zhang X, Su J, Rojas A, Jiang C (2010). Pontine norepinephrine defects in *MECP2*-null mice involve deficient expression of dopamine beta-hydroxylase but not a loss of catecholaminergic neurons. Biochem Biophys Res Commun.

[36] Roux JC, Panayotis N, Dura E, Villard L (2010). Progressive noradrenergic deficits in the locus coeruleus of *MECP2* deficient mice. J Neurosci Res.

[37] Kow LM, Pfaff DW (1987). Responses of ventromedial hypothalamic neurons in vitro to norepinephrine: dependence on dose and receptor type. Brain Res.

[38] Brickley SG, Mody I (2012). Extrasynaptic GABA(A) receptors: their function in the CNS and implications for disease. Neuron.

[39] Farrant M, Nusser Z (2005). Variations on an inhibitory theme: phasic and tonic activation of GABA(A) receptors. Nat Rev Neurosci.

[40] Whissell PD, Lecker I, Wang DS, Yu J, Orser BA (2015). Altered expression of deltaGABAA receptors in health and disease. Neuropharmacology.

[41] Lee CY, Liou HH (2013). GABAergic tonic inhibition is regulated by developmental age and epilepsy in the dentate gyrus. Neuroreport.

[42] Chen L, Chen K, Lavery LA, Baker SA, Shaw CA, Li W, Zoghbi HY (2015). *MECP2* binds to non-CG methylated DNA as neurons mature, influencing transcription and the timing of onset for Rett syndrome. Proc Natl Acad Sci U S A.

[43] Sigel E, Steinmann ME (2012). Structure, function, and modulation of GABA(A) receptors. J Biol Chem.

[44] Mortensen M, Ebert B, Wafford K, Smart TG (2010). Distinct activities of GABA agonists at synaptic- and extrasynaptic-type GABAA receptors. J Physiol.

[45] Korsgaard S, Casey DE, Gerlach J, Hetmar O, Kaldan B, Mikkelsen LB (1982). The effect of tetrahydroisoxazolopyridinol (THIP) in tardive dyskinesia: a new gamma-aminobutyric acid agonist. Arch Gen Psychiatry.

[46] Braat S, Kooy RF (2015). Insights into GABAAergic system deficits in fragile X syndrome lead to clinical trials. Neuropharmacology.

[47] Zhang W, Peterson M, Beyer B, Frankel WN, Zhang ZW (2014). Loss of *MECP2* from forebrain excitatory neurons leads to cortical hyperexcitation and seizures. J Neurosci.

[48] Schwarz LA, Miyamichi K, Gao XJ, Beier KT, Weissbourd B, DeLoach KE, Ren J, Ibanes S, Malenka RC, Kremer EJ, Luo L (2015). Viral-genetic tracing of the input-output organization of a central noradrenaline circuit. Nature.

[49] Lee V, Maguire J (2014). The impact of tonic GABAA receptor-mediated inhibition on neuronal excitability varies across brain region and cell type. Front Neural Circuits.

[50] Weng SJ, Wiggins JL, Peltier SJ, Carrasco M, Risi S, Lord C, Monk CS (2010). Alterations of resting state functional connectivity in the default network in adolescents with autism spectrum disorders. Brain Res.

[51] Smrt RD, Pfeiffer RL, Zhao X (2011). Age-dependent expression of *MECP2* in a heterozygous mosaic mouse model. Hum Mol Genet.

[52] Young JI, Zoghbi HY (2004). X-chromosome inactivation patterns are unbalanced and affect the phenotypic outcome in a mouse model of rett syndrome. Am J Hum Genet.

[53] Wither RG, Lang M, Zhang L, Eubanks JH (2013). Regional *MECP2* expression levels in the female *MECP2*-deficient mouse brain correlate with specific behavioral impairments. Exp Neurol.

[54] Meziane H, Ouagazzal AM, Aubert L, Wietrzych M, Krezel W (2007). Estrous cycle effects on behavior of C57BL/6 J and BALB/cByJ female mice: implications for phenotyping strategies. Genes Brain Behav.

[55] Ure K, Lu H, Wang W, Ito-Ishida A, Wu Z, He LJ, Sztainberg Y, Chen W, Tang J, Zoghbi HY (2016). Restoration of *MECP2* expression in GABAergic neurons is sufficient to rescue multiple disease features in a mouse model of Rett syndrome. Elife.

[56] Bangasser DA, Zhang X, Garachh V, Hanhauser E, Valentino RJ (2011). Sexual dimorphism in locus coeruleus dendritic morphology: a structural basis for sex differences in emotional arousal. Physiol Behav.

[57] Jin X, Li S, Bondy B, Zhong W, Oginsky MF, Wu Y, Johnson CM, Zhang S, Cui N, Jiang C (2016). Identification of a group of GABAergic neurons in the dorsomedial area of the locus coeruleus. PLoS One.

